# Use of tilapia piscidin 3 (TP3) to protect against MRSA infection in mice with skin injuries

**DOI:** 10.18632/oncotarget.4102

**Published:** 2015-05-11

**Authors:** Han-Ning Huang, Yi-Lin Chan, Cho-Fat Hui, Jen-Leih Wu, Chang-Jer Wu, Jyh-Yih Chen

**Affiliations:** ^1^ Marine Research Station, Institute of Cellular and Organismic Biology, Academia Sinica, Jiaushi, Ilan, Taiwan; ^2^ Department of Life Science, Chinese Culture University, Taipei, Taiwan; ^3^ Institute of Cellular and Organismic Biology, Academia Sinica, Taipei, Taiwan; ^4^ Department of Food Science, National Taiwan Ocean University, Keelung, Taiwan

**Keywords:** antimicrobial peptides, tilapia piscidin 3, wound healing, staphylococcus aureus

## Abstract

Antimicrobial peptides (AMPs), represent promising agents for new therapeutic approaches of infected wound treatment, on account of their antimicrobial and wound closure activities, and low potential for inducing resistance. However, therapeutic applications of these AMPs are limited by their toxicity and low stability *in vivo*. Previously, we reported that the 23 amino-acid designer peptide TP3 possessed antimicrobial activities. Here, we analyzed the wound closure activities of TP3 both and *in vivo*. TP3 at doses of up to 40 μg/ml did not affect the viability of baby hamster kidney cells. Furthermore, TP3 was found to be highly effective at combating peritonitis and wound infection caused by MRSA in mouse models, without inducing adverse behavioral effects or liver or kidney toxicity. TP3 treatment increased survival by 100% at 8 days after infection, and accelerated the progression of proliferation, remodeling, and maturation of infected wounds. Taken together, our results indicate that TP3 enhances the rate of survival of mice infected with the bacterial pathogen MRSA through both antimicrobial and immunomodulatory effects. Overall, these results suggest that TP3 may be suitable for development as a novel topical agent for treatment of infected wounds.

## INTRODUCTION

Antimicrobial peptides (AMPs) represent particularly promising agents for new therapeutic approaches of infected wound treatment, as they possess antimicrobial and wound closure activities, and offer little opportunity for the development of resistance [1, 2]. AMPs are short amino acid chain molecules involved in the first line of defense against invading pathogens [3]. In addition to host defense, they are also involved in the modulation of innate immunity [4]. Piscidins are cationic AMPs expressed by fish mast cells [5]. The piscidin family consists of structurally-related mature peptides of 21~44 residues that possess an amphipathic α-helical structure, which suggests that piscidins have bactericidal activities against a variety of microorganisms [6, 7].

Tilapia piscidin 3 (TP3) is an AMP isolated from Nile tilapia (*Oreochromis niloticus*), and was characterized as early as 2012. Tilapia piscidin 3, also known as TP3, is a 23 amino acid peptide that starts with phenylalanine (F) and ends with histidine (H) [8]. TP3 is a pore forming peptide with an α-helix structure, which confers selective cytolytic activity against bacteria. In addition to disrupting bacterial membranes, Tilapia α-helix AMPs have been reported to stimulate immunogenicity, induce a TH1 cellular immune response, and serve as adjuvants for vaccines in fish [9]. TP3 has antimicrobial activity against both Gram-positive and -negative bacteria [8]. Furthermore, clinical case studies have shown that application of AMPs to severely infected cutaneous wounds can clear the infection and improve healing [10]. In addition, previous studies have confirmed that AMPs have immunomodulatory function [11]. A recent study reported that AMPs may promote resistance to bacterial infections by stabilizing the cytoskeleton network in host cells [12]. Thus, TP3 has many features consistent with antibiotics, but potentially has broader applications, and may avoid or reduce concerns of bacterial resistance.

The skin functions as a physical barrier against microbial pathogens. Once this physical barrier is disrupted by wounding, however, microbial pathogens can gain access to tissues [13]. A major problem with skin injuries is the high risk of infection. To treat wound infections, several antibiotic drugs, such as chloramphenicol, gentamicin, neomycin, and bacitracin, are applied topically to open sores. However, the routine use of topical antibiotics leads to the progressive decline of therapeutic efficacies of these antibiotics due to the development of antimicrobial resistance [14-16]. Hence, there is a strong need for the development of new classes of drugs for the treatment of infected wounds.

Treatment with effective antimicrobial agents can both help reduce the risk of infection, and reduce the overall time required for wound healing. Bacteria can colonize wounds within 48 hours after injury, and bacteria such as *Staphylococcus aureus*, *Pseudomonas aeruginosa*, and *Streptococcus spp*. may prolong the inflammatory phase of wound healing [17]. Thus, topical or systematic application of suitable antimicrobial agents may prevent wound infection and/or accelerate wound healing. Inflammation involves the release of biologically active mediators, which attract macrophages and lymphocytes to the wound area; these cells attack foreign debris and microorganisms through phagocytosis [18]. The goal of the current study was to examine the antimicrobial, anti-inflammatory, and wound healing properties of TP3 treatment in MRSA-infected mice. We investigated whether combination treatment of a mouse model with peptide and non-peptide antimicrobial agents can (i) improve the antimicrobial activity of the peptides, (ii) identify novel candidates for antibacterial therapeutic drugs, (iii) inhibit bacterial growth, and (iv) accelerate wound healing.

## RESULTS

### *In vitro* toxicity and efficacy of TP3

**Figure 1:**
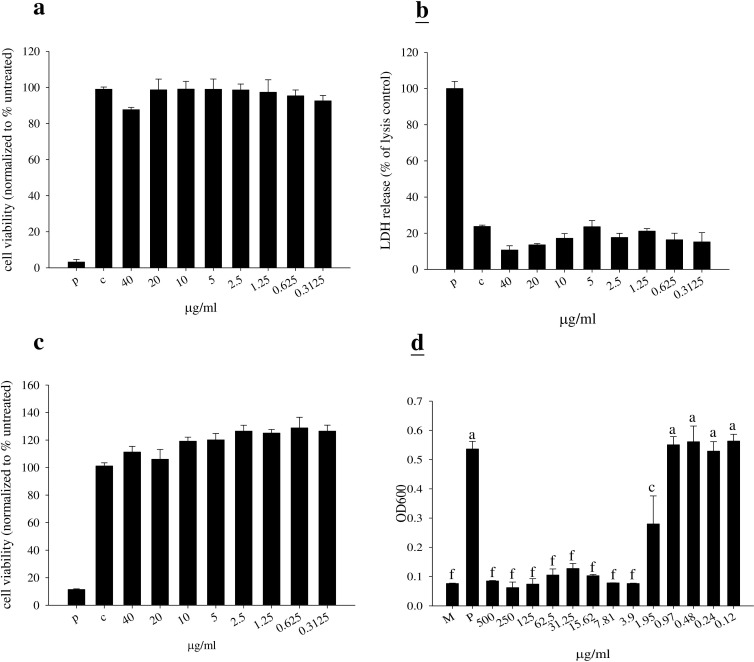
Cytotoxicity of tilapia piscidin 3 (TP3) in baby hamster kidney cells (BHK-21), and antibacterial activity **A.**-**C.** BHK-21 cells were treated with the indicated doses of TP3 for 24 h. Cell viability was measured by neutral red, LDH, and MTT assays. (*R = 3*; *N = 6*). “p” indicates treatment with 10% triton-X-100, “c” indicates no treatment. **D.** MRSA was cultured in the indicated concentrations of TP3. Relative bacterial proliferation was determined based on optical density at 600 nm. (*R = 3*; *N = 6*). Values with different letters show significant differences (*P < 0.05*), as determined by ANOVA. “P” indicates TP3 treatment, “M” indicates treatment with medium alone.

We first studied the cell toxicity of TP3 in BHK-21 cells. Measurement of cell toxicity by neutral red, LDH, and MTT assay revealed that TP3 at various concentrations (up to 40 μg/ml) did not affect cell viability (Figure 1A-1C). The minimal inhibitory concentration (MIC) for TP3 was > 3.9 μg/ml against MRSA. Similarly, > 3.9 μg/ml of TP3 effectively killed MRSA suspended in 10 mM sodium phosphate buffer, pH 7.2 (Figure 1D).

### TP3 does not exert acute toxic effects in mice

Acute toxicity tests for TP3 were evaluated in mice using the up-and-down procedure [19]. Mice received TP3 at a limited dose of 2 mg/mouse via intramuscular (i.m.) injection, and biochemical factors in the blood were subsequently measured. The animals were continuously observed for toxic symptoms for the first 3 hr after dosing. Finally, the number of survivors was noted after 24 hr, and these animals were then maintained for a further 6 days, with observations made daily. Serum samples were collected by tail bleeding at 1, 3, and 6 days. TP3 did not induce any significant changes in the levels of blood urea nitrogen (BUN), creatinine (CRE), total glucose (GLU), or creatine phosphokinase (CPK). On the other hand, glutamic oxaloacetic transaminase (GOT) and glutamic pyruvic transaminase (GPT) were significantly increased at the first day after injection, but over time returned to normal levels (Table 1). Our results suggest that TP3 does not induce systemic toxic effects, even at the highest concentration tested (2 mg/mouse).

**Table 1:** Biochemical parameters of mice after intramuscular injection of TP3 (2 mg/mouse)

Control (n=6)				TP3 (n=6)		
Time (day)	1	3	6	1	3	6
GOT (U/l)	45.6±3.7^A^	41.2±1.5^A^	45.6±3.7^A^	122.6±25.7^C^	47.3±5.9^A^	46±8.08^A^
GPT (U/l)	43.3±5.1^A^	46.4 ±4.3^A^	43.3±5.3^A^	71.3±8.6^C^	36.2±2.7^AB^	35±5.9^AB^
CRE (mg/dl)	0.4±0.1^A^	0.5±0.3^A^	0.5±0.1^A^	0.3±0.05^A^	0.41±0.04^A^	0.54±0.05^A^
BUN (mg/dl)	16.1±1.9^A^	14.2±0.6^AB^	17.3±1.5^A^	15.2±2.4^A^	17.4±2.3^A^	20.2±1.8^AB^
GLU (mg/dl)	216.1±13.2^A^	224.1±21.2^A^	228.1±17.5^A^	258.6±28.2^AB^	219.8±8.2^A^	290.8±53.5^B^
CPK (U/l)	129.3±21.7^AB^	109.1±11.3^A^	100.9±14.7^A^	114±21.9^A^	88.3±5.8^B^	129±15.3^AB^

* All data are expressed as means ± SD and were compared with the ANOVA (n=6).

Differences with *P*<0.05 are considered statistically significant.

### TP3 enhances the survival of mice infected with MRSA and exhibits *in vivo* bacteriostatic properties against MRSA

**Figure 2:**
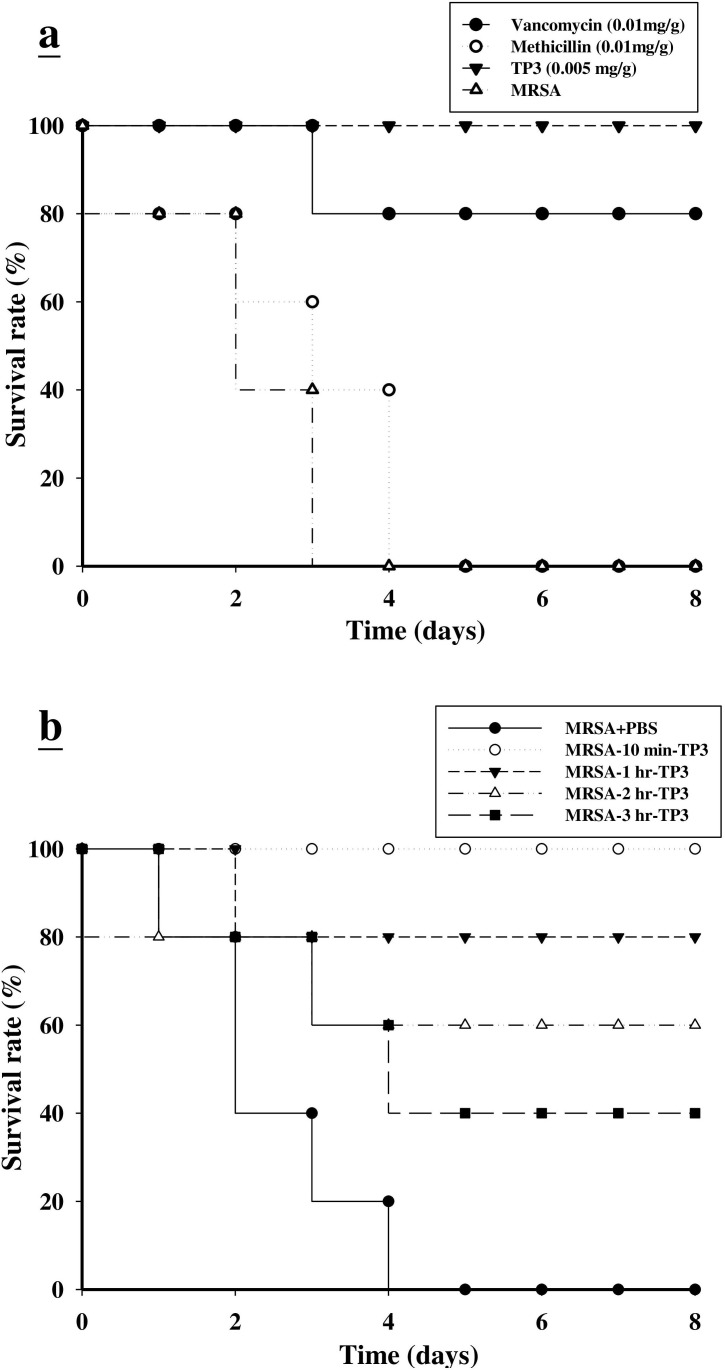
Effects of tilapia piscidin 3 (TP3) treatment on mice infected with MRSA **A.** Mice were injected with MRSA (1×10^6^ CFU/mouse), and independent groups (*N = 5*) were subsequently injected with TP3, vancomycin, and methicillin. The survival rate was monitored on a daily basis for up to 8 days. **B.** To determine the curative potential, mice were first injected with MRSA (1×10^6^ CFU/mouse) and then with TP3 (0.005 mg/g) 10, 60, 120, or 180 min later. At these injection times, the MRSA experimental groups exhibited survival rates of 100%, 80%, 60%, and 40%, respectively.

We proceeded to investigate the bactericidal effects of TP3 *in vivo*, by monitoring the survival of mice infected with MRSA prior to treatment with TP3 or antibiotic. All untreated mice infected with MRSA died within 72 h of infection, whereas co-treatment with TP3 decreased the mortality rate (Figure 2A). At 8 days after MRSA infection, the survival rates were 100%, 80%, and 0% for mice treated with TP3 (0.005 mg/g), vancomycin (0.01 mg/g), and methicillin (0.01 mg/g), respectively. The rates of lethality by 48 h in the untreated groups were 20% in mice infected with MRSA, and treatment with TP3 or vancomycin significantly decreased the rate of mortality (Table 2). Bacteriological evaluation revealed that untreated mice infected with either strain exhibited 100% positive blood cultures and a high level of bacterial colonization (with the numbers of CFU/g being no lower than 10^6^) for all organs tested (Table 2). TP3 treatment significantly reduced the bacterial burden in all examined organs compared to those of untreated controls (*P* < 0.05). These data indicate that TP3 can efficiently control MRSA in the organs of infected mice. To determine the curative potential, mice were first injected with MRSA and then with TP3 (0.005 mg/g) 10, 60, 120, or 180 min later. At these injection times, the MRSA experimental groups exhibited survival rates of 100%, 80%, 60%, and 40%, respectively (Figure 2B). The survival rates of mice treated with TP3 were consistently greater than those of untreated mice (PBS-treated control mice). These data indicate that immediate application of TP3 (0.005 mg/g) is important to prevent severe infection. Application within 10 to 60 min of MRSA infection enabled TP3 to act as an effective curative agent. As such, we proceeded to use TP3 in wound healing infection experiments, and explored antibacterial activity and the promotion of wound repair.

**Table 2:** Effect of TP3, methicillin and vancomycin on mice survival following intraperitoneal injection of 1×10^6^ CFU of MRSA each mouse

Strain and %Treatment	% lethality	Mean ± SD count (CFU/ml)				
		Blood	Peritoneum	Spleen	Liver	Mesenteric lymph nodes
MRSA						
MRSA+PBS	20^B^	5.8×10^7^±1.4×10^7B^	1.x10^10^±3.6×10^9B^	6.8×10^8^±3.x10^8B^	2.1×10^8^±9.2×10^7B^	5.4×10^7^±2.6×10^7B^
MRSA+Methcillin(0.01 mg/g)	20^B^	5.8×10^7^±1.7×10^7B^	4.9×10^9^±2.2×10^9B^	9.5×10^8^±1.9×10^8B^	3.7×10^8^±8.1×10^7B^	6.4×10^7^±5.3×10^7B^
MRSA+Vancomycin(0.01 mg/g)	0^A^	8×10^5^±1.3×10^6B^	2.7×10^9^±1.5×10^9A^	1.8×10^8^±8.4×10^7B^	1.7×10^8^±3.8×10^7B^	2×10^6^±4.4×10^6A^
MRSA+TP3(0.005 mg/g)	0^A^	0^A^	1.4×10^9^±1×10^9A^	9×10^7^±8.2×10^7A^	4.4×10^7^±1×10^7A^	1.6×10^6^±1×10^6A^

Lethality was monitored for 2 day following the injection of TP3 or antibiotic.

### Efficacy of TP3 at promoting *in vivo* wound closure

**Figure 3:**
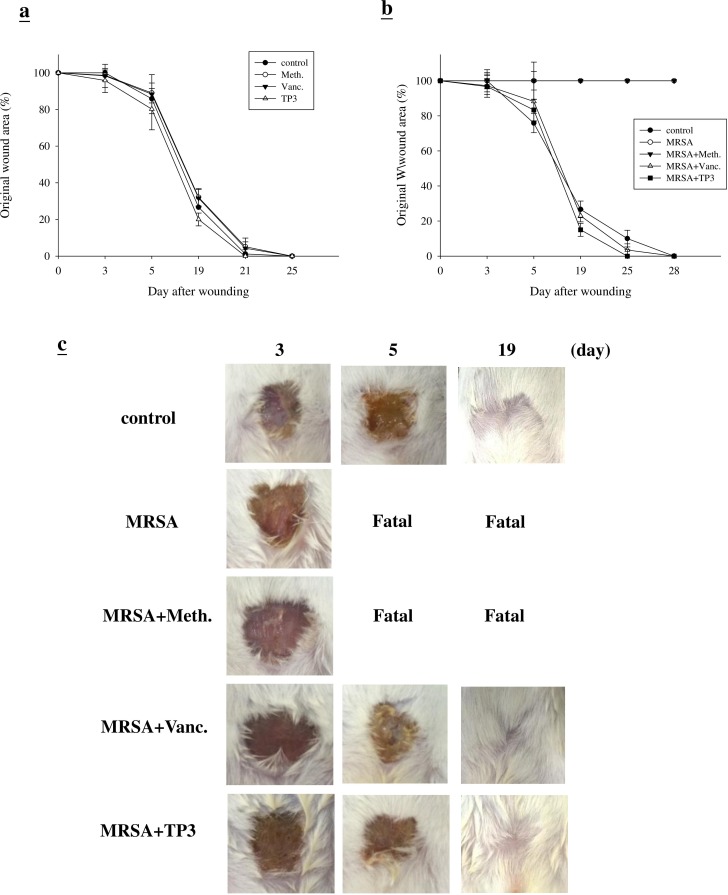
Closure of clean and contaminated wounds The areas of full-thickness wounds (initially 1 cm in diameter) were measured from the time of wounding until closure. **A.** All full-thickness aseptic wounds closed by day 25. Meth., methicillin; Vanc., vancomycin. **B.** Full-thickness wounds contaminated with microorganisms increased in size initially, while TP3 treated wounds did not exhibit the initial expansion and closed somewhat faster (day 25) than vancomycin-treated wounds. **C.** Photographs of representative wounds. “Fatal” indicates that no mice survived.

First, we examined whether TP3 promoted healing of wounds made in an aseptic manner (Figure 3A). We did not observe any statistical difference between the areas of untreated wounds and Tegaderm^TM^ or antibiotic-treated wounds, with all closing by day > 25. This was not unexpected, as skin wounds heal efficiently in healthy mice, and it is unlikely that this process can be significantly improved. However, untreated infected wounds resulted in death in the first week (Figure 3B). Treatment with vancomycin resulted in a similar wound closure time to the control, while wound closure was accelerated by treatment with TP3 alone. Such an increase in wound closure was not observed in uncontaminated wounds, suggesting that TP3 may facilitate wound recovery by combating infection. Unlike the uncontaminated wounds, wound size was largely unchanged after one week in all treatment groups (Figure 3B). By 14 days, wound size in the TP3-treated group was smaller than that of the vancomycin-treated group (*P < 0.05*). However, both groups demonstrated full closure by the end of the 30th day (Figure 3C).

### Microbial loads in treated wounds

**Figure 4:**
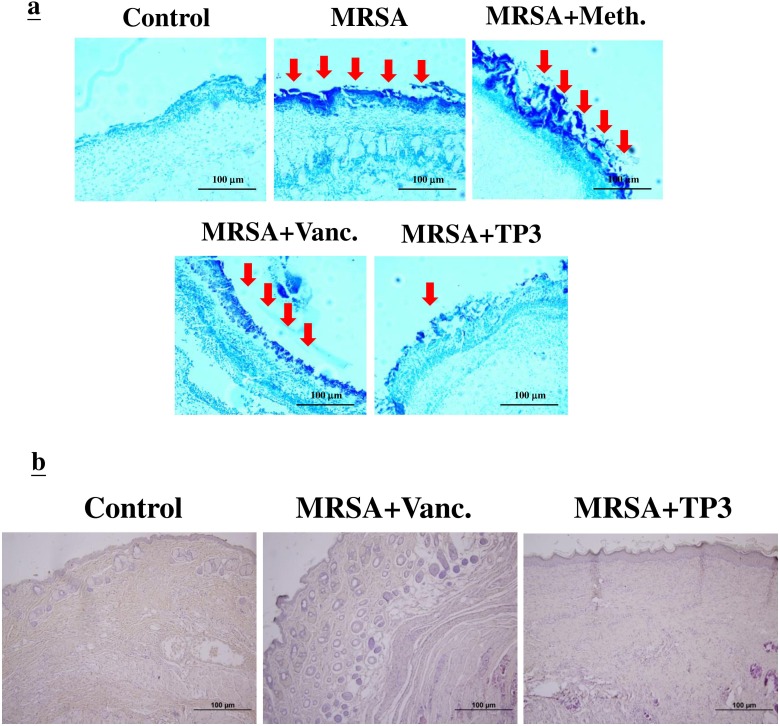
Evaluation of wounds and skin maturation by Gram staining of tissues **A.** Wound biopsy specimens of infected mice (untreated controls or mice treated with the indicated antibiotic or TP3) were Gram stained on day 3. Gram-positive microorganisms are indicated by violet rods. Gram-positive microorganisms were reduced in mice treated with TP3 compared to the untreated group. Arrows indicate Gram-positive microorganisms. The images are representative of two experiments, each performed in triplicate. **B.** Evaluation of dermal and epidermal maturation. Magnification, x100. The length and height of the photomicrographs are 100 μm.

The increase in wound size in untreated contaminated wounds, and the lack of closure in the MRSA and MRSA+Meth (methicillin) treatment groups (Figure 3B), suggested active wound infection. This was supported by quantitative assessment of the wound flora (Table 3). The initial inoculum of approximately 4.5~5.2×10^4^ CFU/10 μl of each organism increased to about 6.7~7.3×10^8^ CFU/10 μl in the MRSA and MRSA+Meth groups by day 3. Between days 3 and 5, the colony counts in the MRSA+Vanc and MRSA+TP3 groups decreased, with the most rapid decrease being observed in the TP3 group (significantly different as compared to the other groups at day 5). In clinical practice, attempts to count MRSA colonies through culturing anaerobes from skin wounds often result in underestimates, due to the aerobic nature of the site. Thus, we evaluated the wounds using Gram staining of tissues, to determine if anaerobes on the skin exceeded the counts achieved by quantitation of aerobes (Figure 4A). Quantitation of the number of Gram-positive organisms per high-power field in the upper dermis reflected the quantitative cell counts. As expected, bacterial loads were reduced more quickly upon treatment with antimicrobial agents.

**Table 3:** Bacterial load. Average of four mice at each time point; control refers to initial inoculation for each mouse

Organism	Condition	Day	Bacterial Count
MRSA	MRSA	0 3 5	4.5×10^4^ CFU/10 ml 7.3×10^7^ CFU/10 ml Fatal
	MRSA+Meth.	0 3 5	5.2×10^4^ CFU/10 ml 6.7×10^7^ CFU/10 ml Fatal
	MRSA+Vanc.	0 3 5 19	4.9×10^4^ CFU/10 ml 3×10^5^ CFU/10 ml 2.6×10^3^ CFU/10 ml 42 CFU/10 ml
	MRSA+TP3	0 3 5 19	4.3×10^4^ CFU/10 ml 1×10^4^ CFU/10 ml 1.42×10^2^ CFU/10 ml 0 CFU/10 ml

### Evaluation of dermal and epidermal maturation

**Figure 5:**
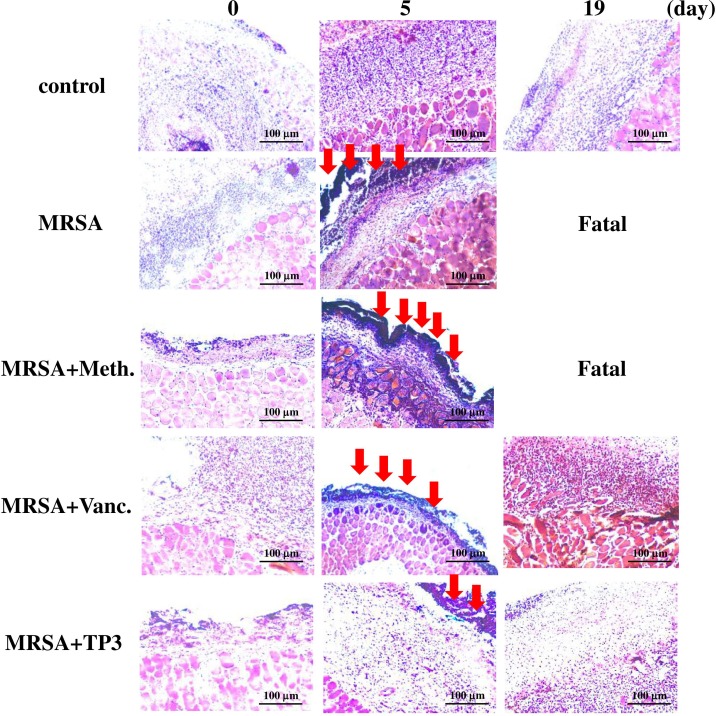
TP3 regulates the accumulation of erythrocytes and platelets in infected wounds A skin region of about 1 square centimeter was removed from the abdomen of non-anaesthetized mice, and the wound was infected with 50 μl of broth mix containing 10^6^ CFU of MRSA alone, or together with methicillin, vancomycin, or TP3. Skin samples from the injured area were fixed and subjected to Giemsa staining at 0, 5, and 19 days post-treatment. Arrows indicate accumulation of immune cells. Magnification, x100. The length and height of the photomicrographs are 100 μm.

The above data demonstrating enhanced wound closure suggest that treatment with TP3 alone facilitate maturation of the dermal matrix. We examined this via routine histological analyses (Figure 4B). Dermal maturation is normally assessed at the proliferation, remodeling, and maturation stages. Wounds treated with TP3 exhibited accelerated progression at all three of these stages. Accelerated healing was also noted in the epidermal compartment (Figure 4B). Wounds treated with TP3 were multilayered as in normal skin, and fully mature by day 25. Keratinization and regeneration of the epithelium showed no signs of irregularity, whereas wounds treated with Tegaderm^TM^ and MRSA+Vanc displayed impairment in overall epidermal maturation as compared to the TP3 group. As such, we proceeded to examine whether TP3 promotes the innate immune response and cytokine production after wound healing in infected mice. Giemsa staining revealed accumulation of immune cells in the skin of infected mice treated with MRSA (Figure 5).

### Mechanism of TP3 activity

**Figure 6:**
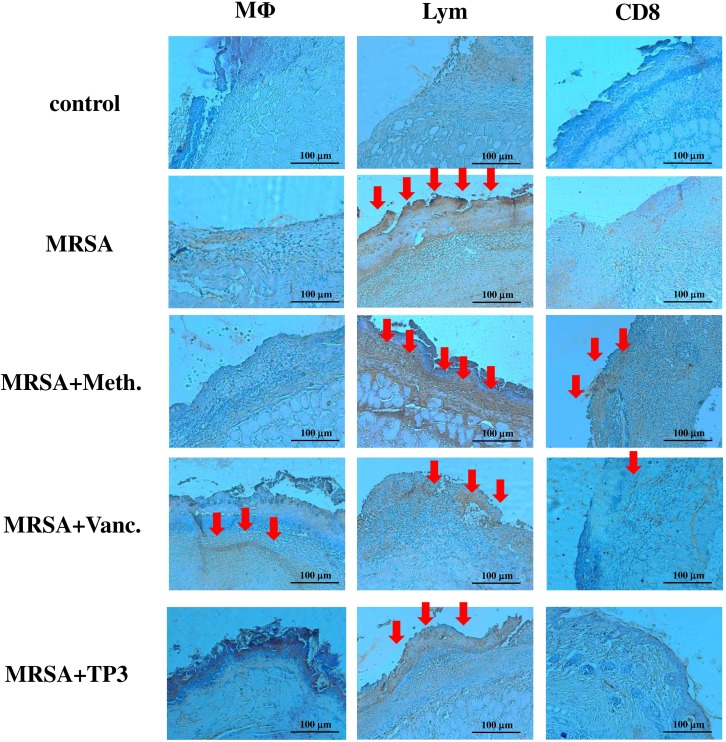
Treatment of infected mice with TP3 enhances infiltration of immune cells Mice were sacrificed at day 3 after wounding. Cryosections of wound sites were fixed in formaldehyde, and immunohistochemical analysis was performed using specific antibodies against macrophages (M), lymphocytes (Lym), or CD8, as indicated. *R = 3*; *N = 3*. Magnification, x100. The length and height of the photomicrographs are 100 μm.

**Figure 7:**
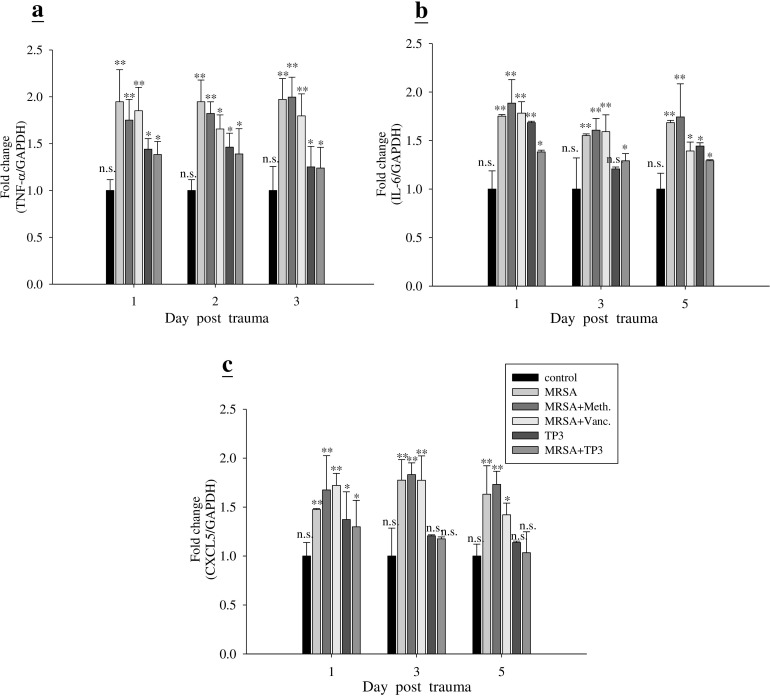
TP3 modulates gene expression profiles in mice Adult mice infected with MRSA were treated with TP3 or antibiotics, while controls were untreated. At different days after infection, total RNA was isolated from the wound and reverse transcribed for use in real-time qPCR analysis of TNF-α, IL-6, and CXCL5 gene expression. *R >* 3; *n >* 3. Values with different letters show significant differences (*P < 0.05*), as determined by ANOVA. *n.s*., not significant; *, significant (*P < 0.05*); **, significant (*P < 0.001*).

Next, we examined the mechanism underlying the direct antimicrobial activity of TP3. The ability of TP3 to modulate the immune cells of mice was measured using IHC (Figure 6) and real-time PCR (Figure 7). IHC with cell surface marker antibodies revealed a significant increase in the infiltration of macrophages, lymphocytes, and CD8 (cytotoxic cells) in infected wounds treated with antibiotic. Such infiltration of immune cells was reduced in the TP3 group (Figure 6), which may be because AMPs directly kill bacteria and thus reduce the area affected by the mouse innate immune response. The pro-inflammatory cytokine IL-6 acts as a potent modulator of innate immunity, while the chemokine CXCL5 enhances the recruitment of macrophages to tissue surrounding wounds [20]. We analyzed wound tissue chemokine and cytokine levels in MRSA-infected mice at 1, 3, and 5 days after treatment. Inflammatory mediators, such as tumor necrosis factor alpha (TNF-α), interferon-γ, interleukin (IL)-6, IL-10, transforming growth factor beta-1 (TGF-β1), and nitric oxide, modulate the wound-healing response [21]. MRSA-infected mice were used as a positive control to confirm cytokine activation. TP3 treatment decreased induction of TNF-α and IL-6. CXCL5 is well known to have chemotactic and activating effects on neutrophils, mainly during acute inflammatory responses [22]. TP3 treatment decreased CXCL5 as compared to the positive controls (Figure 7).

## DISCUSSION

Wound healing involves the precise orchestration of inflammation, epithelialization, tissue granulation, and remodeling. Some AMPs exert antimicrobial effects by directly killing pathogens, or by indirect modulation of the host defense system through enhancing immune responsive cells [23]. Following our previous studies on the effects of AMPs, we examined the suitability of TP3 as a wound-healing agent in a mouse model of MRSA infection. Here, we used a clinically relevant model suitable for elucidating the pathophysiology underlying impairments of wound healing, and for testing novel therapeutic agents. Our study is the first to analyze the toxicity of TP3 *in vitro*. Cell toxicity assays (neutral red, LDH, and MTT assays) were used to reveal that TP3 did not affect cell viability at concentrations of up to 40 μg/ml. Our systemic toxic effect analysis revealed that injection of 2 mg/mouse TP3 does not cause serious side effects in mice. No toxic or other side effects were observed following treatment of mice with TP3, even at relatively high concentrations (2 mg/mouse) within 60 minutes of exposure. TP3 was also demonstrated to have anti-bacterial activity against MRSA *in vitro*, consistent with a previous report that TP3 inhibits bacterial growth [8]. TP3 demonstrated potent bactericidal activity when it was administered to mice after challenge with MRSA. TP3 treatment resulted in 100% clearance of MRSA bacteria from blood after 48 h. Moreover, MRSA CFU decreased in the peritoneum, spleen, liver, and mesenteric lymph nodes after 48 h of TP3 treatment. The data reported here illustrate the potential anti-endotoxin properties of TP3. We used our *in vivo* system to demonstrate that intraperitoneal administration of 0.005 mg/g of TP3 was effective at treating an MRSA infection, increasing the survival rate, and reducing endotoxin and MRSA plasma levels, as compared to antibiotic treatment. Taken together, these findings highlight the utility of TP3 in treating MRSA-infected mice.

We proceeded to evaluate the potential clinical use of TP3 as compared with conventional antibiotics, which are generally the last line of defense against MRSA infections of wounds. Wound healing is associated with a systemic pro-inflammatory state. Our recent clinical work demonstrated that skin leukocyte infiltration is increased during wound healing in patients [24]. Similar to these clinical findings, we observed here increased leukocyte infiltration in the skin of mice during wound healing. This increase was most likely due to increased macrophage infiltration associated with chronic inflammation. Although pre-injury chronic inflammation is deleterious for wound healing, post-injury inflammation, generated through sufficient leukocyte infiltration and cytokine release, is necessary for wound healing. Peptide-based wound healing studies have been reported previously [2], and we applied this platform to demonstrate that TP3 facilitates the healing of infected wounds.

TP3 treatment caused a decrease in TNF-α, IL-6, and CXCL5 at the site of infection on days 1, 3, and 5; on the other hand, MRSA infection induced TNF-α and IL-6. Both Gram-negative (LPS) and Gram-positive (lipoteichoic acid) signature molecules cause up-regulation of pro-inflammatory cytokines through processes that are suppressed by cationic peptides [26]. Cytokines IL-6 and IL-12 play a major role in innate immune activation during wound healing [27]. Accumulation of macrophages and lymphocytes at the wound-healing site produce inflammatory responses, which are mediated by chemokine CXCL5 gene expression [28]. Although TP3 itself caused a modest increase in IL-6 gene expression as compared to the control, this was lower than that induced by MRSA on day 1. The basis for the anti-inflammatory effect of TP3 may be due to contributions from several related mechanisms, including that of IL-10 [23]. Furthermore, TP3 reduced MRSA-induced TNF-α at the wound site on day 1.

Drug development efforts focusing on regulation of the innate defense system have been limited, in part, because of the potential for inducing harmful sepsis responses [29]. Indeed, most antibiotics stimulate the release of bacterial pathogen-associated signature molecule components [30], and thus contribute to the risk of damaging inflammation and sepsis. We have identified that TP3 can directly kill pathogens and reduce inflammation caused by infection, and thereby has potential to provide prophylaxis or treatment of a broad spectrum of infections, while balancing or controlling the attendant inflammatory response. *In vivo* data indicate that TP3 treatment decreases macrophage and lymphocyte recruitment at day 3 after infection. Macrophages have been shown to (i) phagocytose and directly kill bacteria; (ii) deprive bacteria of vital nutrients; (iii) produce cytokines that influence the differentiation state and growth of macrophages themselves and other immune cells; and (iv) recruit other cells. In our study, TP3 induced key chemokines that were likely to be responsible for monocyte and/or macrophage recruitment to the site of infection, and decreased inflammatory cytokines induced by infection.

## CONCLUSIONS

1. The use of TP3 may complement the use of antibiotics. AMPs are unlikely to induce resistance.
2. TP3 is compatible with the use of antibiotics, and does not have any apparent immunotoxic effects. Given the prophylactic efficacy of TP3, and its inability to engender resistance, it may be suitable for situations in which there is a high risk of infection.
3. Our model is valuable for future research on the pathophysiology of wound healing, as well as for testing new therapeutics for the treatment of bacterial infection during wound healing.

## MATERIALS AND METHODS

### Cells and mice

The Baby Hamster Kidney cell line (BHK-21) was cultured in Roswell Park Memorial Institute media (RPMI-1640) supplemented with 10% heat inactivated fetal bovine sera. Balb/c female mice were used for all experiments. All mice were housed in cages under specific pathogen-free conditions, and given water and standard laboratory chow ad libitum during the experiments. All animal handing procedures were in accordance with National Taiwan Ocean University (NTOU) guidelines. All procedures were approved by the Animal Care and Use Committee of NTOU.

### Reagents

Hematoxylin-eosin (H&E) (Cat no. 105175, Merck, Darmstadt, Germany) and Giemsa stain solution (Cat no. 51811826, Sigma, MO, USA) were used for histological staining. Antibodies against macrophages (Cat no. 550282, BD Biosciences, CA, USA), lymphocytes (CD3e) (Cat no. 550277, BD Biosciences, CA, USA), and CD8a (Cat no. 14008182, eBiosciences, CA, USA) were used for immunohistochemistry (IHC).

### *In vitro* toxicity

Cells were cultured at a density of 5×10^4^ cells per well in flat-bottomed 96-well plates, and supplemented with various combinations of TP3. After 24h, cell viability were measured with neutral red uptake assay [31], Cytotoxicity Detection Kit (LDH) (Roche Applied Science, Indianapolis, IN, USA), and CellTiter 96 Aqueous One Solution (Promega, Madison, WI, USA), following the vendor's instructions.

### Synthesis of tilapia piscidin 3 peptides and bacteriostatic analysis

Peptides were synthesized by GL Biochem (Shanghai, China) using a solid-phase procedure of Fmoc chemistry. Crude peptides were extracted, lyophilized, and purified by reverse-phase high-performance liquid chromatography (HPLC). The molecular masses and purities of the purified peptides were respectively verified by mass spectroscopy and HPLC. Synthetic peptides at > 95% purity were reconstituted in phosphate-buffered saline (PBS; pH 7.4) for the experiments. The TP3 sequence was FIHHIIGGLFSVGKHIHSLIHGH.

Minimal inhibitory concentrations (MICs) were determined using standard protocols [32]. For MIC assessment, compounds were diluted to final concentrations of 500, 250, 125, 62.5, 31.25, 15.62, 7.81, 3.9, 1.95, 0.97, 0.48, 0.24, and 0.12 μg/ml. Twenty microliters of each dilution was mixed in a microtiter plate well with 20 μl of the appropriate bacterial indicator suspension and 160 μl of TSB for *S. aureus* to a total volume of 200 μl. Three replicates were examined for each *S. aureus* strain, compound, and concentration. Positive controls contained water instead of compounds, and negative controls contained compounds without bacterial suspensions. Microbial growth was automatically determined by optical density measurement at 600 nm (Bioscreen C; Labsystem, Helsinki, Finland). Microplates (catalog no. 3599; Corning, NY, USA) were incubated at 37°C. Absorbance readings were taken at hourly intervals over a 24-h period, and the plates were shaken for 20 s before each measurement. The experiment was repeated twice. The lowest compound concentration that resulted in zero growth by the end of the experiment was taken as the MIC.

### *In vivo* toxicity

To determine the toxicity of TP3, TP3 was dissolved in phosphate-buffered saline (PBS; pH 7.4) and administered as intramuscular bolus injections in the left thigh (2 mg/mouse). Mice were observed for signs of systemic toxicity. To study the effect of treatment on biochemistry, mice (*n* = 6 in each group) were treated with PBS (control). Blood samples (0.2 ml) were collected on days 1, 3, and 6 after the final injection of TP3, and used to determine the serum levels of glutamic oxaloacetic transaminase (GOT), glutamic pyruvic transaminase (GPT), blood urea nitrogen (BUN), creatinine (CRE), total glucose (GLU), and creatine phosphokinase (CPK).

### Therapeutic use in a mouse model of MRSA sepsis

Female Balb/c mice (6-8 weeks old) were injected intraperitoneally with 10^6^ CFU MRSA per mouse. Ten minutes after MRSA injection, mice were injected intraperitoneally with vancomycin (0.01 mg/g mouse body weight), methicilin (0.01 mg/g mouse body weight), or TP3 (0.005 mg/g mouse body weight). In a second set of experiments, mice were given intraperitoneal injections of TP3 (0.005 mg/g mouse body weight) at 10, 60, 120, or 180 min after MRSA injection. The survival rate and status were recorded every 24 h for up to 192 h. To examine bacterial dissemination, mice were sacrificed at 48 h after injection with antibiotics or TP3, and the bacterial numbers in blood, peritoneum, spleen, liver, and mesenteric lymph nodes were recorded. Colony counts from the diluted bacterial solutions were expressed relative to those at the start of treatment. These experiments consisted of four groups, and each group contained 5 mice.

### Mouse models for wound healing

Female Balb/c mice (6-8 weeks old) were used for wound healing experiments. All mice were housed individually to prevent fighting and further damage to the wounds, and they were provided with food and water ad libitum. Mice were maintained on a 12h light: dark cycle at room temperature, and acclimatized to the environment for at least a week before use in experiments. All researchers wore caps, sterile gloves, gowns, and shoe covers when handling mice. Hair was removed from the back of the mice by shaving, and a full thickness wound (1 cm in diameter) was then created in the exposed region. Each wound was inoculated with 50 μl of broth mix containing 10^6^ CFU (colony forming units) of MRSA. At 5 min after inoculation, 50 μl TP3 (2 mg/ml dissolved in phosphate-buffered saline [PBS]) were applied to the wound. Thirty minutes after treatment, wounds were covered with Tegaderm (3M, St. Paul, MN) to maintain uniformity, and to prevent the mice from removing the treatments. Based on initial experiments, we examined the wounds at 0, 3, 5, and 19 days post-injury, so as not to disturb the infection [27]. Such examinations captured the transitions from inflammatory to regenerative, and regenerative to resolving phases of wound healing [28]. Animals were subsequently euthanized by CO_2_ inhalation and the wounds assessed. Four individuals in each group were examined at each time point for each experiment. Each wound was measured and then removed from the animal, with unwounded skin taken from the contralateral dorsum as a control. Each biopsy was bisected with three sections being used for tensiometry and histology, and two sections for quantitative determination of microbial load. Wound healing studies were repeated in triplicate.

### Wound closure measurements

Tracings were taken immediately after injury. For uncontaminated wounds, wound size was determined every second day. For contaminated wounds, mice were euthanized at days 3, 5, and 19, and tracings of the wound edges were made. Wound areas were determined using the Macintosh Adobe Photoshop program, Histogram Analysis. The percentage of wound contraction was calculated as follows: % Wound contraction = (A_0_ – At)/A_0_ × 100, where A_0_ is the original wound area, and At is the area of wound at the time of biopsy (every two days) [30].

### Assessment of wound infection

Multidrug-resistant strains of *Staphylococcus aureus* (MRSA) commonly associated with human wound infections were selected to generate a polymicrobial solution. The MRSA strain is a clinical isolate from stool obtained from Taipei City Hospital (Heping Fuyou branch) [33]. The initial inoculum was prepared by culturing aerobic bacteria in Tryptic Soy Broth (TSB) overnight at 37°C. Broths were subsequently centrifuged at 1000 rpm for 15 min, and resuspended in TSB with 15% glycerol, or chopped meat extract with 15% glycerol (for aerobic bacteria). The concentration was adjusted to 10^6^ CFU/50 μl, and stored at −80°C. Prior to wound application, the bacterial stocks were re-mixed. Microbial load was determined by direct plating, followed by freeze-thaw and CFU enumeration, in parallel with inoculations. The inoculum was delivered by sterile pipettes to the center of open wounds. After euthanasia (at day 0, 3, 5, or 19), two bisected tissue segments were used to determine microbial load using the protocol for human wound biopsy culture, as stated in the UPMC Clinical Microbiology Laboratory Procedure Manual. Tissue biopsies were weighed and placed in 1.5 ml of TSB, and then homogenized in a tissue grinder. A single drop of the homogenate was placed on the slide and Gram stained for rough assessment (if one or more bacteria are present within the oil immersion field, the expected count in the tissue is at least 10^5^ CFU/g). Serial dilutions (1:10 [0.1 μl original solution plus 0.9 μl distilled water]) of the tissue homogenate were made using distilled water. The CFU/g of tissue was calculated as follows:

CFU/g = plate count (1/dilution) × 10/wt. of homogenized tissue.

### Immunohistochemistry (IHC)

Skin tissues were removed and fixed as previously described [34]. In brief, the cryosections were fixed with 4% formaldehyde, and the tissue samples were stained with hematoxylin/eosin, Giemsa, or Gram stain. IHC was analyzed by three independent investigators. Images were taken using a BX-51 microscope (Olympus, Japan).

### Isolation of messenger (m)RNA and real-time PCR

Total RNA was isolated from wound tissues and purified using a Qiagen RNeasy kit. Reverse transcription into cDNA was performed with iScript cDNA Synthesis Kits (BIO-RAD, USA) according to the manufacturer's recommendations. Real-time polymerase chain reaction (PCR) analysis was used to analyze gene expression, according to the manufacturer's instructions. The iQSYB^®^ Green Supermix (BIO-RAD, USA), 0.5 ml of cDNA, and 500 nM of specific primer pairs were used for selected genes, and a primer pair for GAPDH was used for the reference. Quantitative PCR was performed under the following conditions: 40 cycles of 1 min at 95°C, 30 s at 55°C, and 1 min at 72°C. The threshold cycle number (Ct) was calculated with BIO-RAD software. Relative transcript quantities were calculated using the ΔCt method with GAPDH as the internal reference gene. ΔCt is the difference in the threshold cycles of messenger (m)RNA for selected genes relative to those of GAPDH mRNA. Real-time PCR was performed in triplicate for each experimental group.

Primer sequences:

TNF-α: F-GGTGTTCATCCATTCTCTAC
R-CCCAGCATCTTGTGTTTC
IL-6: F-TCCATCCAGTTGCCTTCTTG
R-TTTCTCATTTCCACGATTTCCC
CXCL5: F-CTGACCCCAGTGAAGATAAG
R-CCGATAGTGTGACAGATAGG
GAPDH: F-ACAATGAATACGGCTACAG
R-GGTCCAGGGTTTCTTACT

### Statistical analysis

The experiments were conducted with three or more replicates, and repeated at least three times. Error bars represent the standard deviation, and significant differences between groups (*P < 0.05*) were determined using analysis of variance (ANOVA). Different letters above the bars were used to indicate significant differences between groups. Histological and *in vivo* study results were representative of three independent experiments. A group of 7 mice was used for each treatment, and each experiment was repeated three times.
